# Similar Metabolic Costs for Mangrove Tree Crabs (*Aratus pisonii*) in Historic and Range‐Shifted Habitats

**DOI:** 10.1002/ece3.71882

**Published:** 2025-07-24

**Authors:** Blaine D. Griffen, Zachary J. Cannizzo, Laura S. Fletcher, Shelby N. Gold, Bailey N. Marlow, Hannah C. Richardson, Rocky L. Seeley, Amanda C. Dominguez Villalobos

**Affiliations:** ^1^ Biology Department Brigham Young University Provo Utah USA; ^2^ National Oceanic and Atmospheric Administration Office of National Marine Sanctuaries—National Marine Protected Areas Center Silver Spring Maryland USA

**Keywords:** climate‐induced range shift, energetics, life history variation, metabolic rate, Q_10_, range expansion

## Abstract

Climate‐induced range shifts may displace species into novel habitats where their life history characteristics may differ in response to new physiological conditions. One such species is the mangrove tree crab, *Aratus pisonii* , that has expanded beyond mangrove habitats into salt marshes, with the help of anthropogenic structures such as boat docks that mimic its natural habitat in many ways. Individuals in the salt marsh grow to smaller sizes and have different reproductive patterns than individuals in the native mangrove or in boat dock habitats. We examined the metabolic rates of crabs associated with each of these three habitats to determine whether changes in energy expenditure could account for the life history changes that have been documented. We found that the metabolic patterns were similar in the three habitats, with metabolic rate increasing with body size and with temperature, being higher for females than for males and increasing during reproduction. However, once these factors were accounted for, there was no additional difference in metabolic patterns between habitats. Combining these patterns with known patterns of temperature differences and differences in food intake between the mangrove, salt marsh, and boat docks provides mechanistic insight into the energy mismatch that has been created by this range expansion from mangroves to salt marshes. The energy dynamics in these different habitats are consistent with and are capable of explaining the observed patterns of life history variation that accompany this range expansion. Our study provides an example of a mechanistic approach to understanding the influence of climate change and associated range shifts on life history variation across habitat types.

## Graphical abstract

We show that metabolic costs of mangrove tree crabs are similar in native mangrove habitats and in salt marshes that have recently been colonized with climate change. Our results suggest that changes to life history of this species that accompany this range expansion are the result of energetic constraints in salt marshes.

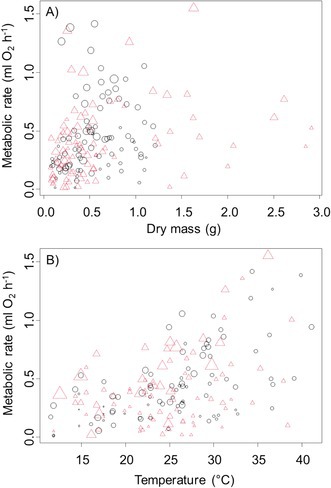

## 1 Introduction

Climate change is causing species to shift their geographic ranges in response to changing environmental conditions in order to remain within niches that are suited to their physiology. These shifts have largely occurred towards higher latitudes and higher altitudes (e.g., de la Fuente et al. 2022), and are occurring in terrestrial and aquatic systems alike (Chen et al. 2011; Poloczanska et al. 2013). These climate‐induced range shifts can result in both species loss in lower latitude regions of their historic ranges (Franco et al. 2006; Thomas et al. 2006; Larsen et al. 2011; Wiens 2016), and expansions towards the poles as cold temperatures in higher latitude areas become more moderate (Thomas 2010; Essl et al. 2019; Pinsky et al. 2020).

Climate‐induced range expansions can alter numerous aspects of ecological systems, but one of the most consequential changes may occur when the ranges of existing community members shift at different rates, resulting in a reshuffling of ecological communities (Alexander et al. 2016; Tomiolo and Ward 2018), as is expected to occur with existing bird assemblages in California (Stralberg et al. 2009) and with odonate species in northern Europe (Pélissié et al. 2022). This reshuffling can alter trophic interactions and food web dynamics (Pinsky et al. 2020) and can expose individual species to novel biotic and abiotic conditions, including thermal regimes (e.g., Cannizzo and Griffen 2019). Such changes have far‐reaching consequences for individual behavior, physiology, and life history when compared to conditions in the historic range.

One such scenario is occurring on the southeast coast of the United States in mangrove and salt marsh ecosystems. Mangrove forests are migrating northward along the US Atlantic coast at rates ranging from 13 to 45 km per decade, depending on the species (Williams et al. 2014), as wintertime freezes abate at the northern edge of their range. Mangroves are ecosystem engineers that support a broad range of species, both in their canopy and among their prop roots (Hogarth 2007). However, not all species that inhabit mangrove forests are moving at the same rate. The mangrove tree crab *Aratus pisonii* is a neotropical arboreal crab that inhabits mangroves from Brazil to Florida (Rathbun 1918). The northward range expansion of this species, in response to climate change, has outpaced that of its mangrove habitat, and this species has now taken up residence in salt marsh ecosystems that occur north of the mangrove range limit (Riley et al. 2014).

Changes in behavior, morphology, and physiology have accompanied this habitat transition. Behaviorally, crabs in the mangrove occupy a home tree as a base from which to forage in the mangrove canopy and among prop roots and sediment when tidal conditions allow, with the frequency of movement decreasing exponentially with distance from their home tree in a Lévy walk fashion (Cannizzo and Griffen 2016). In salt marshes, crabs do not use either a home base or a Lévy walk foraging pattern (Cannizzo and Griffen 2016), thus altering the pattern of foraging impacts (Griffen et al. 2017). Additionally, crabs are more likely to engage in high‐risk ritualistic displays on salt marsh substrate than mangrove (Cannizzo et al. 2019). Along with these life history changes also come substantial morphological changes that have reproductive consequences. Specifically, mangrove tree crabs living in salt marsh ecosystems grow to smaller sizes, mature at smaller sizes, and show altered reproductive patterns when compared to their mangrove counterparts that occur further to the south (Riley and Griffen 2017). In addition, when compared to their counterparts in mangrove habitats, mangrove tree crabs in salt marshes store less energy (Cannizzo et al. 2018) and produce lower quality offspring (Cannizzo et al. 2020).

This species also commonly occupies boat docks embedded within salt marshes throughout its expanding range. Boat docks apparently serve as isolated habitats that mimic the native mangrove habitat. Crabs on boat docks have body sizes, physiological condition, and reproductive patterns that match those found in crabs from the mangroves much more than those found in crabs from the marsh surface surrounding the boat docks (Cannizzo et al. 2020). This may reflect thermal patterns (Cannizzo and Griffen 2019) and diet patterns (Cannizzo et al. 2018) on boat docks that mimic those in mangrove habitats. Consequently, boat docks appear to act as stepping stones that are facilitating the northward range expansion of this species.

The decrease in body size along a northward gradient exhibited by this species is opposite that expected based on Bergmann's rule (Bergmann 1848), where body size is expected to increase with latitude, and differs from that of other coastal crab species (e.g., *Uca pugnax* ; Johnson et al. 2019). There may be multiple mechanisms leading to this decrease in size within salt marsh habitats. First, reduced body size may reflect energy limitations due to reduced food quality in salt marsh habitats. Cannizzo et al. (2020) examined the diet of crabs in mangrove and salt marsh habitats and found that crabs in the salt marsh generally consume a lower quality diet than those in the mangrove. Second, reduced body size may reflect reduced growth due to energy limitation in warmer conditions, as more energy is expended on basal metabolic costs (Teplitsky and Millien 2014). The historic limitation of mangrove tree crab movement into salt marshes appears to reflect the occurrence of wintertime freezing events (Cannizzo and Griffen 2019). However, during summer months, salt marshes present a thermal challenge as crabs spend much more time exposed to direct sunlight due to the lack of canopy shading that occurs in the mangroves (Cannizzo et al. 2018). This exposure to direct sunlight should result in higher temperature‐dependent metabolic rates in this poikilotherm. However, local adaptation to thermal conditions has been demonstrated across a broad range of organisms, including marine invertebrates with widely dispersing larvae (Sanford and Kelly 2011). Thermal adaptations, such as the documented behavior of crabs dipping in water during high tide (Cannizzo et al. 2018), may ameliorate the impacts of increased thermal exposure in salt marsh habitats, but could also lead to other costs such as increased risk of predation.

In addition to temperature, several other factors may also influence the metabolic rates of crabs. Specifically, metabolic rates increase strongly with body size (Griffen and Sipos 2018), can differ across sexes (Griffen et al. 2024), are often altered during reproduction, both for gravid (Stancil et al. 2024) and vitellogenic individuals (Smith et al. 2022), and may also be influenced by injury via limb loss (Fletcher et al. 2022; Smith et al. 2022; Griffen et al. 2024).

To test the hypothesis that the smaller body size, lower energy storage, and altered life history characteristics of crabs in the salt marsh can be explained by higher metabolic energy expenditure than for those found in the mangroves, we performed in situ metabolic rate measurements of mangrove tree crabs collected from both habitats along the Florida coast. In addition, we also measured metabolic rates of crabs collected from dock structures within salt marsh habitats, as these anthropogenic structures provide both physical conditions similar to mangroves (vertical structure with abundant shade) and improved foraging opportunities for mangrove tree crabs (Cannizzo et al. 2018) while also enhancing reproductive performance in ways that may have facilitated the northward range expansion of this species (Cannizzo et al. 2020).

## 2 Methods

We measured metabolic costs in two separate studies, referred to here as “Experiment 1” and “Experiment 2.” Experiment 1 examined the metabolic rates of crabs at different times of the year to capture the influence of air temperature across a wide range of values, as well as incorporate any other potential seasonal shifts that may occur because of changes in forage or other factors. In addition, in Experiment 1 we measured the metabolic rates of crabs collected from mangrove habitats, from salt marshes, and from boat docks located within salt marsh habitats. Only non‐gravid crabs were used in this experiment. In contrast, Experiment 2 focused on a single period of time to remove environmental (temperature) differences, but instead focused on the various individual factors that may influence the metabolic rate (reproductive state, injury level, parasite infection, and whether a crab had just molted). Additionally, Experiment 2 focused solely on crabs from the mangrove and salt marsh habitats. Both experiments examined a wide range of crab sizes reflective of the size of individuals found in the respective habitats.

### 2.1 Experiment 1

We measured the metabolic rates of *A. pisonii* collected from mangroves (Pepper Park: 27°29′42.00″N 80°18′12.00″W; Round Island Park: 27°33′33.00″N 80°19′53.00″W; Oslo Riverfront Conservation Area: 27°35′12.25″N 80°21′54.44″W), salt marsh (Guana‐Tolomato‐Matanzas National Estuarine Research Reserve: 30° 0′49.00″N 81°20′42.00″W), and boat docks (Palm Valley boat launch:, Boating Club Road: 30° 7′58.18″N 81°23′5.87″W; Vilano Beach: 29°55′13.69″N 81°18′0.36″W). Samples were taken on 15 different days in each habitat type beginning in May 2016 and concluding in February 2017 to encompass the full range of temperatures experienced throughout the year (Table 1). The total number of crabs sampled and measured from each habitat type was 60, 60, and 53 crabs from mangroves, boat docks, and the salt marsh, respectively. We measured both adult males and non‐gravid adult females of a range of sizes representing the size distribution of the population from each habitat. We placed crabs into 125 mL glass jars that had a hole drilled in the lid. We included a control jar that had no crab because the oxygen probe used for measurements slowly depleted oxygen throughout a trial. We measured oxygen concentrations using an Apogee Instruments MO‐200 Oxygen meter (0.1% resolution) that was inserted through the hole in the lid. Both the gap around the probe and the lid of the jar were then sealed with putty and petroleum jelly, respectively.

**TABLE 1:** Numbers of crabs sampled per month in each habitat type for Experiment 1. Samples were collected on multiple days within most months, with an average of 3.8 ± 3.2 crabs sampled and measured on any single day.

Month	Mangrove	Dock	Salt marsh
January	—	—	—
February	9	7	—
March	—	—	—
April	—	—	—
May	7	—	—
June	5	8	7
July	3	2	7
August	—	1	1
September	9	2	3
October	—	4	8
November	9	1	6
December	18	9	21

Animals were left for 5 min to calm down following placement into experimental chambers and before the start of the trial. Metabolic rates of crabs can be elevated following capture and handling, and can remain elevated for more than 12 h (Wilson et al. 2021). The impacts of handling have not been measured on metabolism of *A. pisonii* , and while our 5‐min acclimation period may have been insufficient to return metabolism to resting levels, all crabs were treated in an identical manner, facilitating comparison of relative metabolic rates across crabs within our study. Any potential interactions between stress responses and other aspects of individual variation (e.g., reproductive state, parasite infection, time of year, habitat, etc.) could potentially contribute to metabolic patterns, but is not detectable with our experimental procedures. Further, there are fundamental differences in the amount and type of food consumed by crabs in the different habitats (Cannizzo et al. 2018), and diet intake can strongly influence metabolic expenditure (i.e., the influence of diet amount and type on specific dynamic action, reviewed in Secor 2009). One of our goals was therefore to include the impacts of this differential consumption in our metabolic rate measurements, and this was only possible if rates were measured soon after capture rather than transporting crabs to the lab and waiting for the stress of capture to subside.

Air temperature at the time of each measurement was obtained using a thermometer placed next to the jars, while barometric pressure was obtained from the nearest weather station using publicly available data. Each trial was run for 2 h, with oxygen concentration and temperature being recorded every 10 min throughout that time. This provided sufficient data for analysis but was not long enough to result in hypoxic conditions. We then used the following equations provided by the manufacturer of the oxygen probe to first apply a temperature correction Equation (1) and then to determine the amount of oxygen consumed by applying the ideal gas law Equation (2).

(1) $$O_{T}=\left(\frac{T_{c}+273.15}{T_{m}+273.15}\right)\times O_{m}$$

(2) $$O_{F}=3200\times \left[\left(\frac{O_{T}}{100}\right)\times \left(\frac{P\times V}{R\times T}\right)\right]$$

Where *O*_*T*_ is the temperature corrected reading, *T*_*c*_ and *T*_*m*_ are the air temperature at calibration and at the time of the trial, respectively, *O*_*m*_ is the oxygen reading provided by the instrument, *O*_*F*_ is the mass of oxygen consumed, *V* is the volume of the experimental container, *P* is the air pressure at calibration, *R* is the ideal gas constant, and *T* is the temperature at the time of measurement in Kelvin.

We made these calculations for the experimental and control chambers separately at each time point and determined oxygen consumption as the slope of the relationship between oxygen concentration throughout the 2‐h trial. To correct for oxygen consumption by the probe, we subtracted the oxygen consumption in the control chamber from the consumption in the experimental chamber.

Graphical analysis suggested nonlinear impacts of both body mass and temperature on metabolic rate. We therefore analyzed Experiment 1 by fitting 5 different models to the data, and then comparing these models using AIC to select the best‐fitting model. Each model used respiration rate (mL h^−1^) as the response variable. We used generalized additive models with a gaussian distribution so that nonlinearity could be incorporated with the use of smoothed nonparametric terms without the need to explicitly describe the form of that nonlinearity mathematically. The first model included temperature as a fixed variable and body mass as a smoothed variable to account for nonlinearity. The second model included body mass as a fixed variable and experimental temperature as a smoothed variable to account for nonlinearity. The third model included both body mass and experimental temperature as smoothed variables to account for nonlinearity in both of these variables. The fourth model again included both body mass and experimental temperature as smoothed variables, but also included the interaction between these variables as a fixed term to account for interactions between these two variables. The fifth model again included both body mass and experimental temperature as smoothed variables, but also included a three‐way interaction between body mass, temperature, and sex. This three‐way interaction was significant. Therefore, to more clearly understand our results, we analyzed the data using the model selection procedure described above (using models 1–4) for males and females separately. In addition, we also compared metabolic rates for crabs sampled from boat docks, mangroves, and salt marshes using an ANOVA.

Lastly, we also used these data to approximate the Q_10_ of *A. pisonii* , or the increase in metabolic energy expenditure with a 10° increase in temperature. Because we did not control temperature and each trial was conducted at a different temperature, we made this approximation using a regression approach (Griffen and Sipos 2018). We first fit a polynomial model to metabolic rate (mL O_2_ h^−1^) as a function of body mass, including both the linear and quadratic terms of the predictor to account for nonlinearity. We then took the residual from this analysis (metabolic rate once body mass was accounted for) and used a linear regression to examine the change in residual metabolic rate with experimental temperature. We used the parameter estimate (± SE) as an approximation of the Q_10_.

### 2.2 Experiment 2

We measured the metabolic rates of *A. pisonii* collected from mangroves at Pepper Park, FL (27°29′41.67″N 80°18′12.14″W) on August 5, 2022, and from a salt marsh in Anastasia State Park, FL (29°52′40″N 81°16′32″W) on August 6, 2022, following methods given by Fletcher et al. (2022). We collected adult crabs of both sexes by hand (*n* = 100 from each habitat type). Collected crabs were immediately placed into experimental chambers for measuring metabolic rate. We made metabolic rate chambers using 150‐mL syringes with the tip sealed using silicone and with an 8‐mm hole drilled in the top of the barrel through which gas samples were extracted.

After placement into the chamber, individuals were given 5 min before experimentation began. During the duration of the trial, crabs were placed in the shade and were left undisturbed. Air temperature, barometric pressure, and humidity were measured next to the chambers every 10 min using a BTMeter (Model 100‐AAP). We added 0.5 mL of water to each chamber to ensure that water vapor was saturated throughout the trial. The volume of the chambers was varied by altering the plunger of the syringe so that larger crabs had larger volumes of air (range 40–90 mL). This was necessary, based on preliminary trials, to ensure that a measurable change in oxygen occurred during the trial. Differences in volume were accounted for in the calculations of metabolic rate for each crab.

At the start of each trial, the port in the barrel of the chamber was sealed using a septum designed for headspace gas analysis (Bridge Analyzers Incorporated, model #001620). Crabs were then left undisturbed for the duration of the trials. Crabs were visually inspected discretely, so as not to disturb the crabs, at 10‐min intervals to determine the activity level of each crab. All crabs remained inactive throughout the trials. The average trial duration was 1 h 26 m, though the duration varied slightly between crabs due to the time it took to measure the gas in each chamber. The precise duration of the trial was included in the calculation of metabolic rate for each crab.

At the conclusion of a trial, a needle was inserted through the septum and a gas sample was withdrawn to measure the concentration of oxygen and carbon dioxide using a multi‐gas oxygen probe from Forensics Detectors (Model #FD‐600, 0.01% resolution). Air samples were withdrawn at 0.5 L min^−1^ using the built‐in pump. Each individual crab was measured only once. At the conclusion of the trial, each crab was placed into an individual plastic bag and was immediately placed on ice. Crabs were then shipped on dry ice to Brigham Young University in Provo, UT where they were stored for approximately 1 month at −80°C before dissection.

Crabs were dissected by first determining whether the carapace was soft, indicating a recent molt. Next, we measured the volume of the crab via water displacement using a graduated cylinder and noted any missing or regenerating limbs and whether the crab was gravid. For females, we then removed the dorsal carapace and examined the ovaries under a dissecting microscope to determine whether the crab was vitellogenic. For each crab, we also counted the number of acanthocephalan parasites found within the carapace. Each crab was then placed in a pre‐weighed boat and dried to constant weight at 65°C and was weighed to 0.01 mg using a Mettler Toledo DualRange scale (Model number XS205).

We used Equation 4.4 from Lighton (2019), given here as Equation (3), for determining metabolic rate (VolO_2_) under static conditions:

(3) $$\mathrm{Vol}\;O_{2}=\frac{V\left(F_{i}O_{2}-F_{e}O_{2}\right)-F_{e}O_{2}\left(\mathrm{Vol}\;H_{2}O\right)}{1-F_{e}O_{2}\left(1-\mathrm{RQ}\right)}$$

where *V* represents the volume of the gas in the chamber (determined by subtracting the volume of the crab from the chamber volume), FᵢO_2_ and F_e_O_2_ are the initial and final concentrations of oxygen in the chamber measured with the oxygen probe, VolH_2_O is the change in the volume of water vapor throughout the trial (set to zero because we added 0.5 mL of water to each chamber to ensure water vapor saturation), and RQ is the respiratory quotient, or the amount of carbon dioxide produced in relation to the amount of oxygen consumed. This value usually falls between 0.7 and 1.0. We used a value of 0.85 because *A. pisonii* is known to be omnivorous (Erickson et al. 2008), because its diet differs with habitat (Cannizzo et al. 2018), and because this middle‐of‐the‐road value limits error to 3% (Vleck 1987). Finally, we adjusted the metabolic rate to standard temperature and pressure using Equation 2.1 from Lighton (2019).

Given that this second set of measurements was conducted over just two days, the range of experimental temperatures was minimal (i.e., 30°C–33°C). We therefore examined the impacts of body mass and temperature on metabolic rate (mL h^−1^) using a linear model, with temperature and body mass treated as fixed variables and with a squared polynomial term for body mass to account for nonlinearity in its impact. We additionally included the number of limbs that were missing and the number of limbs that were regenerating in this analysis, since these are not independent of body mass (i.e., limbs that are missing will reduce body mass, and this would be somewhat corrected by limbs that are regenerating). We then used the “step” function in the base R package to reduce this model to the best‐fitting model. Next, we controlled for differences in metabolism due to body mass by taking the residual of this best‐fitting model (i.e., metabolism after controlling for any differences due to body mass) and using this residual metabolic rate for all other comparisons. This included comparing residual metabolic rates between sexes, between gravid and non‐gravid females, between vitellogenic and non‐vitellogenic females, between crabs that did or did not have a soft carapace, and between crabs sampled from the mangrove and from the salt marsh using separate *t*‐tests for each. We also conducted a two‐way ANOVA to determine if the impacts of vitellogenesis and being gravid were additive, based on the presence of an interaction. Finally, we used a linear model to examine residual metabolic rate as a function of the number of acanthocephalan parasites.

## 3 Results

### 3.1 Experiment 1

**FIGURE 1:**
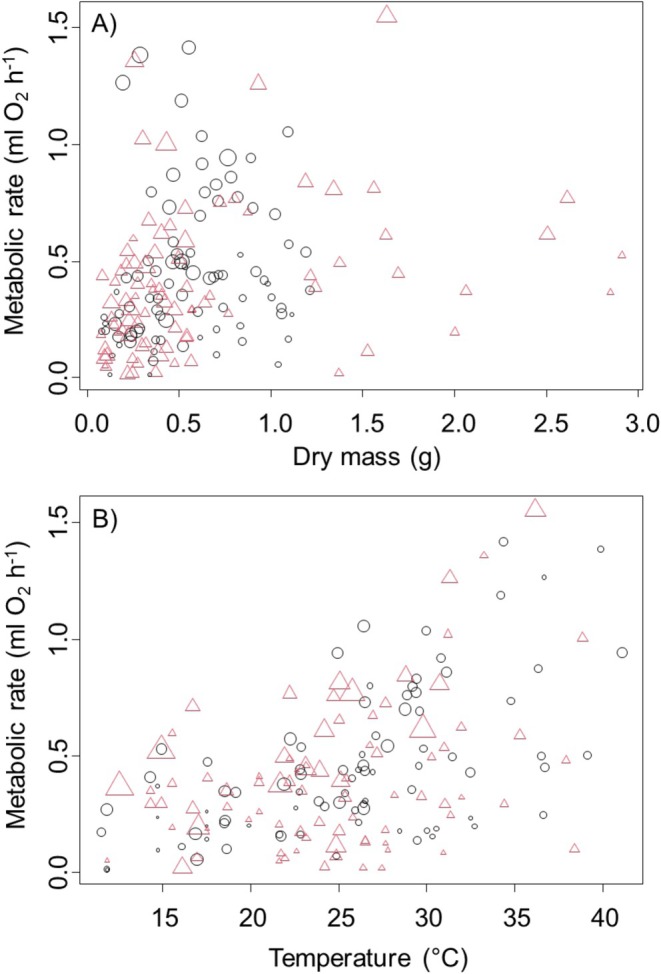
Data from Experiment 1 crabs combined. Black circles show females; red triangles show males. (A) Metabolic rate of *Aratus pisonii* as a function of body mass with relative temperature shown as symbol size. (B) Metabolic rate as a function of temperature, with relative body mass shown as symbol size.

Experiment 1 was conducted at different times during the year and therefore included a range of ambient temperatures. When the data for males and females were analyzed together, we found that there was a significant interaction term between body size, temperature, and sex (*t* = 3.31, *p* = 0.001). In order to better understand these results, we therefore analyzed the two sexes separately. For females, we found that all four models provided equally good fit to the data (delta AIC of all models was within 0.6). Based on Occam's Razor, we therefore selected the simplest model, which was model 1. Based on this model, metabolic rate increased nonlinearly with body mass (smoothed term in GAM, *F* = 8.27, *p* = 0.005, Figure 1A) and metabolic rate also increased by 0.027 ± 0.004 mL O_2_ h^−1^ for each 1°C increase in temperature (parametric term in GAM, *t* = 7.05, *p* < 0.0001, Figure 1B). Overall, this model explained 39.6% of the deviance in metabolic rates.

For males, the best model was model four (delta AIC for next best model was 14.64, model 3). Based on this model, we found that metabolic rates of male *A. pisonii* increased nonlinearly with both body mass (smoothed term in GAM, *F* = 3.01, *p* = 0.009, Figure 1A), and while the smoothed term for temperature was included in the model, it was not significant (*F* = 1.27, *p* = 0.332, Figure 1B). However, we found that the interaction between the impacts of body mass and temperature was significant (*t* = 3.50, *p* = 0.0008), and it appeared that temperature had the strongest impact on the largest individuals (symbol sizes in Figure 1). We found no difference in metabolic rate based on sex (*t*‐test, *t* = 0.12, *p* = 0.91). Finally, we also found no difference in metabolic rates for crabs collected from boat docks, mangrove habitats, or salt marshes (ANOVA, *F* = 0.82, *p* = 0.44). Lastly, we found that the Q_10_ of *A. pisonii* is 1.23 ± 0.03 (*t* = 7.82, *p* < 0.0001).

### 3.2 Experiment 2

**FIGURE 2:**
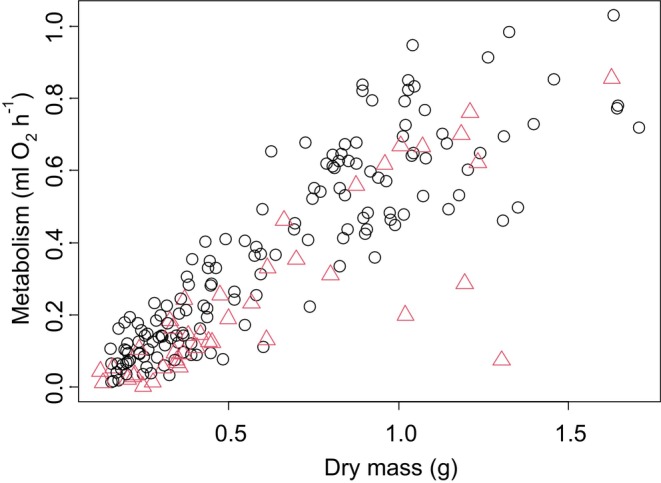
Data from Experiment 2 crabs combined. Black circles show females; red triangles show males. Metabolic rate of *Aratus pisonii* as a function of body mass.

**FIGURE 3:**
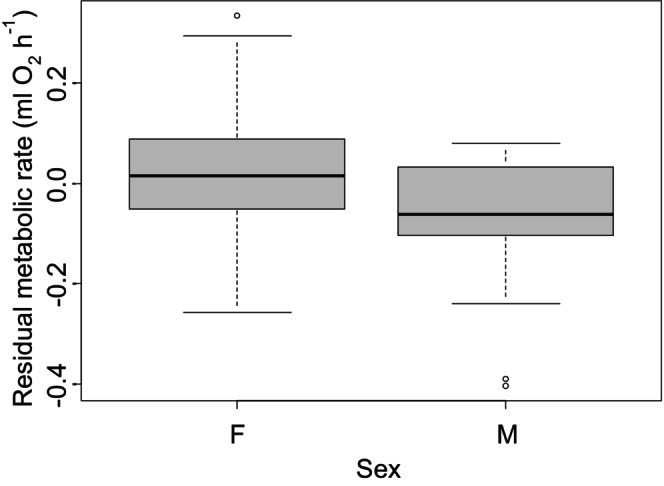
Residual metabolic rate, after accounting for differences with body mass, from Experiment 2 for female and male crabs.

**FIGURE 4:**
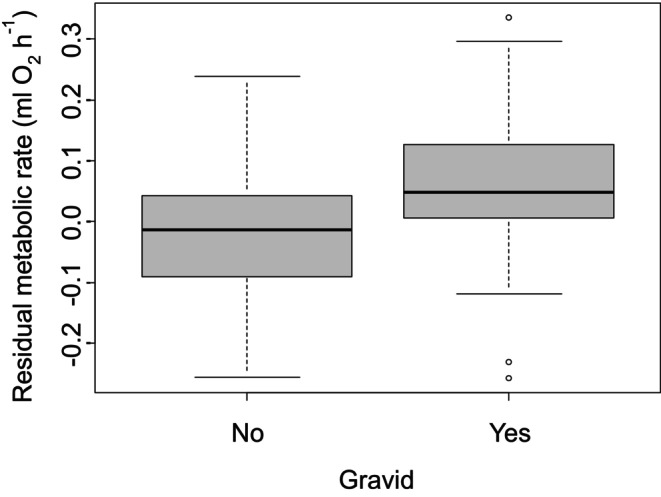
Residual metabolic rate, after accounting for differences with body mass, from Experiment 2 for gravid and non‐gravid female crabs.

**FIGURE 5:**
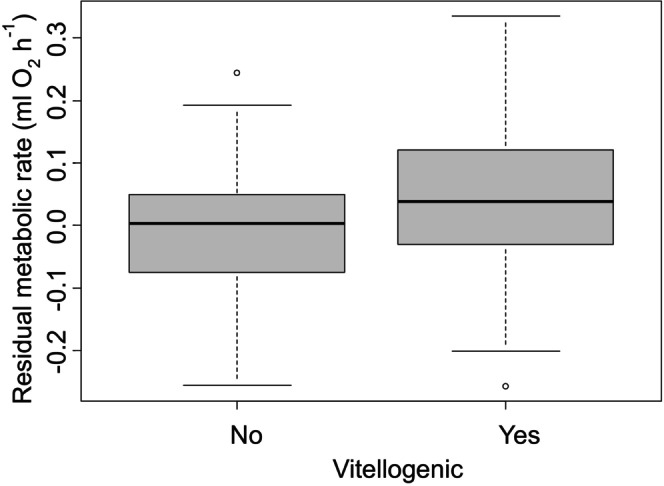
Residual metabolic rate, after accounting for differences with body mass, from Experiment 2 for vitellogenic and non‐vitellogenic female crabs.

**FIGURE 6:**
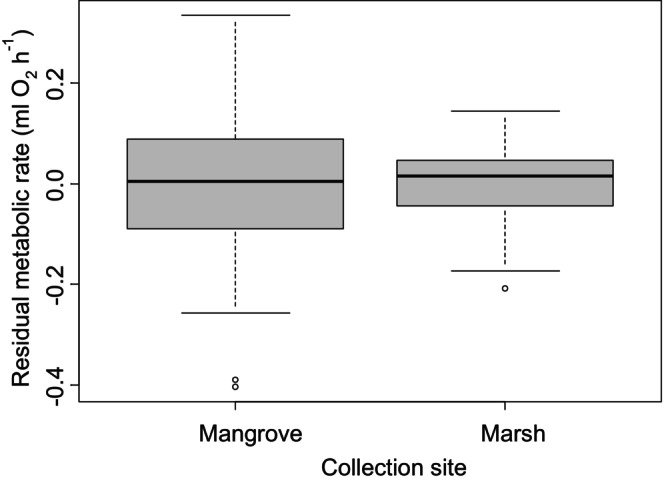
Residual metabolic rate, after accounting for differences with body mass, from Experiment 2 for crabs collected from the mangrove forest and those collected from the salt mars.

Experiment 2 examined crabs at a single point in time rather than at different times of the year. We found that the metabolic rate of crabs increased nonlinearly with body mass. Specifically, there was an initial metabolic increase of 1.00 ± 0.09 mL O_2_ h^−1^ for every 1‐g increase in dry body mass (*t* = 10.89, *p* < 0.0001, Figure 2); however, this effect of increasing body mass decreased towards larger body sizes (squared term in polynomial regression, *t* = −4.53, *p* < 0.0001, Figure 2). In addition, metabolic rate increased by 0.02 ± 0.01 mL O_2_ h^−1^ with each additional limb that was missing, though this effect was marginally insignificant (*t* = 1.76, *p* = 0.08) and decreased by 0.02 ± 0.01 mL O_2_ h^−1^ with each additional missing limb that was regenerating, though this effect was again marginally insignificant (*t* = −1.95, *p* = 0.053). In this experiment, we found that mean residual metabolic rates of females (after accounting for impacts of body mass) were 0.076 mL O_2_ h^−1^ higher than for male crabs (*t*‐test, *t* = 3.95, *p* = 0.0002, Figure 3). We also found that reproduction increased the metabolic rates of females. Specifically, we found that metabolic rates of gravid females were 0.084 mL O_2_ h^−1^ higher than for non‐gravid females (*t*‐test, *t* = 4.88, *p* < 0.0001, Figure 4) and that metabolic rates of vitellogenic females were 0.058 mL O_2_ h^−1^ higher than non‐vitellogenic females (*t*‐test, *t* = 3.22, *p* = 0.002, Figure 5). Thirty‐six crabs were simultaneously vitellogenic and gravid, and these two processes had additive impacts on metabolic costs (ANOVA, vitellogenic: *F* = 12.51, *p* = 0.001, gravid: *F* = 22.77, *p* < 0.0001, interaction term: *F =* 1.87, *p* = 0.17). We found no difference in metabolic rates between crabs that did or did not have soft carapaces (*t*‐test, *t* = 0.29, *p* = 0.78) or between crabs collected from mangroves and crabs collected from salt marshes (*t*‐test, *t* = 0.18, *p* = 0.86, Figure 6).

## 4 Discussion

We used two sets of measurements to examine the metabolic rates of the mangrove tree crab, *Aratus pisonii* , collected from mangrove, salt marsh, and boat dock habitats. We have shown that metabolic rate increased with body mass and with temperature, as expected. In addition, we found that metabolic rate was higher for females than for males, and when compared at a single point during the summer, we found that gravid and vitellogenic females both have higher metabolic rates than nonreproductive females. Finally, after differences in body mass and temperature were accounted for in the statistical analysis, we found no evidence from either of our experiments of an underlying difference in metabolic rates for crabs based on habitat type. These results have important implications for our understanding of the life history changes that are associated with the climate‐induced range expansion of this species from mangroves to salt marshes.

The metabolic response of *A. pisonii* appears to be similar to that of other brachyuran crab species. In a meta‐analysis of metabolic rates across brachyuran crabs, Griffen and Sipos (2018) found that the Q_10_ for brachyuran crabs as a whole is 1.26, similar to the 1.23 ± 0.03 found here. *A. pisonii* in mangroves generally remain under the mangrove canopy, spending just a fraction of their time (~1%) in direct sunlight, while individuals in the salt marsh lack a solar refuge and spend a much more variable amount of time in direct sunlight, averaging nearly 50% of the time (Cannizzo et al. 2018). Further, summertime temperatures in the salt marsh are ~10°C warmer than in the shade at the same location (Cannizzo et al. 2018). Combining the Q_10_, the difference in temperature when exposed to direct sunlight, and the time exposed to direct sunlight, this suggests that a crab may expend roughly 12% more energy to meet basal metabolic costs in the salt marsh than a similarly sized conspecific in the mangrove, simply because of the temperature differences that they experience. This difference in energy expenditure, combined with the generally lower quality of food consumed in the salt marsh (Cannizzo et al. 2018), may explain the lower energy stored in the hepatopancreas of salt marsh crabs compared to their mangrove counterparts (Cannizzo et al. 2018). This in turn may help explain the prevalence of smaller body sizes in the salt marsh, as lower energy reserves mean less energy available for growth.

These patterns of relative metabolic costs of life in the salt marsh vs. in the mangrove could also potentially help explain differences in reproductive patterns in the two habitats. Crabs in the salt marsh produce clutches of eggs more frequently, but each clutch is approximately half as large as clutches of crabs in the mangrove, even after standardizing for differences in body size (Riley and Griffen 2017). Further, *A. pisonii* uses a mixed capital‐income breeding strategy, where initial clutches of the year in early spring are energetically financed using stored energy reserves, while clutches produced later in the year during the summer are produced using energy brought in through foraging during the reproductive process (Carver et al. 2021). The increased energy expenditure in warmer salt marsh conditions may hasten the depletion of stored capital energy, thus hastening the transition from capital to income breeding as the breeding season progresses and may limit the ability to allocate energy to egg production, thereby decreasing the size of clutches that are produced, consistent with observed patterns. The elevated metabolic rates of vitellogenic and gravid crabs demonstrated here indicate that reproduction will further exacerbate the drawdown of energy reserves, even after the costs allocated directly to egg production. This should further reduce the capability of energetically stressed crabs in the salt marsh to reproduce. The metabolic impacts on growth and reproduction hypothesized in these last two paragraphs could be examined more quantitatively using optimal bioenergetics modeling, following approaches used by Fletcher and Griffen (2024).

After statistically accounting for differences in body size and temperature, we found no differences in the metabolic rates of crabs collected from different habitats. This suggests that differences in body size and life history across habitats that have previously been documented are not due to local adaptations for energy expenditure in the different habitat types, but likely stem from differences in temperatures to which animals in the different habitats are exposed, as described above, combined with differences in the nutritional quality of food consumed in the different habitats (Cannizzo et al. 2020).

The patterns described here are consistent with patterns seen in other ectotherms as temperatures increase. For instance, the sea urchin *Letechinus variegatus* experiences increases in metabolism with temperature that outstrip increases in feeding rates, leading to energy shortfalls as temperatures increase (Lemoine and Burkepile 2012). More broadly, ectotherms commonly experience reduced aerobic scope as warming temperatures approach their thermal limit (Pörtner 2002), and this can lead to energy limitation and constraints on the ability to cope with stressful conditions (Sokolova 2013). These general patterns and the consistent data on *A. pisonii* suggest that as this species continues to expand its range into salt marsh habitats, we may expect a continued shift in life history to reflect strategies that may be optimal under energetic mismatches associated with warming conditions that stem from a lack of shade and poorer food options in the salt marsh. Additionally, these results further bolster the finding that boat docks and other anthropogenic structures that provide islands of shade and improved foraging opportunities within salt marsh habitats (Cannizzo and Griffen 2019) serve as an important refuge for *A. pisonii* , facilitating its northward range expansion.

## Author Contributions

**Blaine D. Griffen:** conceptualization (lead), data curation (lead), formal analysis (lead), funding acquisition (lead), investigation (lead), methodology (lead), project administration (lead), supervision (lead), visualization (lead), writing – original draft (lead). **Zachary J. Cannizzo:** data curation (equal), investigation (equal), writing – review and editing (equal). **Laura S. Fletcher:** investigation (equal), writing – review and editing (equal). **Shelby N. Gold:** investigation (equal), writing – review and editing (equal). **Bailey N. Marlow:** investigation (equal), writing – review and editing (equal). **Hannah C. Richardson:** investigation (equal), writing – review and editing (equal). **Rocky L. Seeley:** investigation (equal), writing – review and editing (equal). **Amanda C. Dominguez Villalobos:** investigation (equal), writing – original draft (equal).

## Conflicts of Interest

The authors declare no conflicts of interest.

## Supporting information


**Data S1.** Aratus base analysis_new calculations.

**Data S2.** Respiration data.

## Data Availability

All data associated with this manuscript are included as [Link], [Link].
